# Can Buprenorphine Be Overdosed? The Ceiling Effect and Its Clinical Implications

**DOI:** 10.3390/ph19060903

**Published:** 2026-06-06

**Authors:** Rozalia Beata Kozińska, Julia Ślęzak, Ignacy Ilski, Maja Podsiadła, Kamil Biedka, Emilia Królewicz

**Affiliations:** 1 Scientific Society for Psychopharmacology, Department of Forensic Medicine, Wroclaw Medical University, Mikulicza-Radeckiego 4J, 50-345 Wroclaw, Poland; julia.slezak@student.umw.edu.pl (J.Ś.); ignacy.ilski@student.umw.edu.pl (I.I.); maja.podsiadla@student.umw.edu.pl (M.P.); 2 Department of Physiology and Pathophysiology, Division of Pathophysiology, Wroclaw Medical University, Chalubinskiego 10, 50-368 Wroclaw, Poland; kamil.biedka@umw.edu.pl; 3 Department of Medical Biochemistry, Wroclaw Medical University, 50-368 Wroclaw, Poland; emilia.krolewicz@umw.edu.pl

**Keywords:** buprenorphine, ceiling effect, buprenorphine overdose, polysubstance use, opioid overdose, respiratory depression

## Abstract

Opioids comprise a class of substances characterized by a narrow therapeutic window and significant euphoric effects. Opioid-related mortality remains a critical global health issue, primarily resulting from respiratory depression associated with full μ-opioid receptor agonists. Buprenorphine is distinguished by its pharmacological profile as a partial μ-opioid receptor agonist, exhibiting a ceiling effect on both respiratory depression and euphoria. These characteristics render buprenorphine particularly valuable in opioid replacement therapy for opioid use disorder and in the management of both chronic and acute pain. Current literature indicates that, due to these properties, buprenorphine possesses a superior safety profile compared to other opioids. This narrative review evaluates whether buprenorphine can cause fatal overdose despite its protective ceiling effect and examines cases of buprenorphine-related fatalities. The review synthesizes data from pre-clinical, clinical, pharmacological, and toxicological studies. Current literature supports the consensus that the established ceiling effect on respiratory depression does not fully eliminate the risk of fatal overdose. Polysubstance use, particularly co-ingestion of other central nervous system depressants, significantly increases this risk, and most buprenorphine-related deaths involve such combinations. Additionally, buprenorphine’s safety is reduced in vulnerable populations, including opioid-naive individuals, children, and patients with pre-existing respiratory compromise. While buprenorphine alone presents a substantially lower fatality risk compared to other opioids, the ceiling effect is pharmacodynamic rather than absolute, and toxicity or fatal overdose may still occur. Current data gathered from clinical and pre-clinical studies suggest that buprenorphine is safer than full μ-opioid receptor agonists, but it is not without risk. The ceiling effect’s protective benefits depend on context. Therefore, despite its improved safety profile, buprenorphine should not be regarded as risk-free.

## 1. Introduction

Opioids represent one of the most frequently misused drug classes [1]. Since 2010, opioid-related mortality has risen substantially in developed countries [2]. The primary risk associated with opioid abuse is the narrow therapeutic index of full mu-agonists, which significantly increases the probability of an overdose [3].

Respiratory depression is the leading cause of death in opioid overdose, resulting from opioid binding to receptors in the preBötzinger complex and Kölliker-Fuse nucleus within the medullary network [4,5]. Each opioid exhibits a distinct receptor affinity, which determines its capacity to activate or inhibit neuronal signaling pathways essential for respiratory function. Opioids, which are full agonists, like morphine and fentanyl, bind and activate these central nervous system receptors more efficiently than buprenorphine. Consequently, all effects, both desirable and undesirable, like analgesia, euphoria, and respiratory depression, are greater than in the case of buprenorphine. This relationship underscores the need to understand individual opioid receptor mechanisms to more accurately predict their impact on respiratory function, and by extension, their overdose mortality rate [4,6].

Buprenorphine is a semisynthetic opioid and a thebaine derivative. Its unique pharmacological properties provide a safety profile superior to that of other opioids [7]. Clinical indications include chronic and acute pain management, as well as opioid replacement therapy [8,9,10]. Buprenorphine is suitable for use in high-risk populations, including the elderly, individuals with renal impairment, and patients with opioid dependence [11]. Unlike most opioids, buprenorphine acts as a partial agonist at the mu-opioid receptor (MOR) and as an antagonist at kappa (KOR) and delta (DOR) receptors. It also exhibits activity at the nociceptin/orphanin FQ receptor (NOR) [12,13,14].

In 1966, buprenorphine was synthesized at the Reckitt & Colman research facility (Hull, England), headed by John Lewis. Such a drug became the result of years of research concerning a search for an analog of classic opioids with lower addictive character and no respiratory depressive effects. Buprenorphine began to be widely used as a medical treatment in the transitional period between the 70s and 80s as an analgesic drug with agonist-antagonist activity. Further extensive testing carried out, among other places, at the Addiction Research Center (ARC) helped characterize its effects. It was only in 1978 when a paper by Jasinski et al. [15] indicated that due to buprenorphine’s pharmacology (partial agonism of μ-opioid receptor and long-lasting action), the substance had to play an important role in contemporary treatment of opioid addiction. In the early 21st century, the introduction of public–private partnerships and the development of legal frameworks have facilitated a revolution in healthcare. From restrictive medical practice, care has evolved into a more flexible form of substitution therapy tailored to each patient [16]. Innovation in the development of buprenorphine preparations allows for the fine adjustment of the treatment regimen in cases of chronic pain and OUD. Transdermal and buccal drug delivery systems are crucial for managing pain because of their high absorption rates and consistent pharmacokinetics, thereby ensuring patient safety. When it comes to treating addiction disorders, the innovation of extended-release medications (injections, implants) is of major importance, since it has led to higher compliance among patients [17]. Historically, inclusion of naloxone into buprenorphine medications was considered as a deterrent to non-oral abuse. This assumption was supported by the supposition that naloxone, as a powerful μ-receptor antagonist, causes immediate withdrawal reactions when attempting injection. Nevertheless, the current pharmacological studies undermine the efficiency of this practice. It can be explained by the fact that the binding potency of buprenorphine to the opioid receptor is ten times higher than that of naloxone, and the short half-life of this compound makes it pharmacologically inactive during substitution from agonists. Though this compound may prevent the rapid development of euphoria, it does not provide strong clinical support as an absolute drug to avoid parenteral and inhalation routes of administration. Moreover, there are certain risks associated with the effects of repeated naloxone administration. Specifically, long-term administration of this compound can lead to upregulation of opioid receptors; therefore, increasing sensitivity to lethal overdose after termination of treatment becomes a problem [18].

In pharmacology, the ceiling effect describes the phenomenon in which increasing a drug’s dose no longer enhances its therapeutic effect, as the maximal effect plateaus [19,20]. Buprenorphine’s low intrinsic efficacy at the MOR limits its ability to activate G-protein signaling. Human volunteer studies have demonstrated that increasing buprenorphine doses produces a plateau in adverse effects, while plasma concentrations rise linearly, confirming a pharmacodynamic ceiling rather than an absorption limit [21]. This partial agonism explains the ceiling observed in respiratory depression and euphoria, but not in analgesia, where receptor reserve remains sufficient to achieve complete pain relief [22]. These observations have led to the hypothesis that buprenorphine may act as a self-antidote, restricting its own toxicity and that of co-administered full mu-agonists through pharmacological ceiling effects and receptor blockade [23].

In toxicology and pharmacology, overdose refers to an event of the administration of a substance that exceeds the threshold at which the organism can safely metabolize or tolerate, leading to adverse physiological reactions, clinically significant symptoms beyond the intended therapeutic effect [24,25]. Fatal overdose refers to an event resulting in death attributable to the direct pharmacological consequences of the substance. In the case of opioids, the most common effects are: irreversible respiratory depression, hypoxia, cardiorespiratory arrest, and end-organ failure [24,26]. Moreover, toxicity refers to the extent to which a given substance can harm an organism by reflecting the pharmacological and biochemical mechanisms by which it disrupts normal physiological functions [27]. Poisoning is a broader term referring to a clinical state resulting from harmful exposure to a drug, causing organ dysfunction or systemic harm. It can be intentional, accidental, or iatrogenic [28]. However, poisoning does not necessarily imply ingestion of a supratherapeutic dose; harm may result from a standard dose in vulnerable populations [29].

The aim of this review is to investigate the implications of buprenorphine’s ceiling effect by analyzing preclinical and clinical evidence related to its receptor pharmacology, dose–effect relationships, and respiratory effects. More importantly, cases of death involving buprenorphine ingestion are evaluated in order to explain the relationship between the ceiling effect and buprenorphine overdose fatalities, with particular focus on drug interactions involving central nervous system depressants. The review aims to explain the pharmacological basis of the ceiling effect and to critically assess the hypothesis that buprenorphine cannot result in fatal overdose due to the ceiling effect.

## 2. Methods

This article constitutes a narrative review. A comprehensive literature search was conducted. Relevant publications were identified by searching three major electronic databases: PubMed, Embase, and Web of Science. The following search terms were applied individually and in Boolean combinations using AND/OR operators: buprenorphine, buprenorphine overdose, buprenorphine toxicity, ceiling effect, opioid overdose, respiratory depression, opioid use disorder, pharmacodynamics, withdrawal, and polysubstance use. No date-range restriction was imposed; all publications available from inception through March 2026 were considered eligible for inclusion. 

Given the broad and heterogeneous nature of the research question, publications were selected based on their relevance to the pharmacology, toxicology, and clinical implications of buprenorphine’s ceiling effect, encompassing preclinical animal studies, controlled human volunteer pharmacodynamic studies, randomized and non-randomized clinical trials, retrospective postmortem toxicological analyses, case reports, case series, systematic reviews, and previously published narrative reviews. The emphasis was on articles from the last 5 years; however, given the specificity of this manuscript’s subject, a few older publications were included. The rationale for choosing older publications was to capture the original studies on the pharmacology of buprenorphine. Publications older than 5 years were included only if their content contributed significantly to the subject of this manuscript and no newer studies on the same topic existed. Study selection was performed iteratively by the authors through screening titles and abstracts, followed by full-text review of potentially relevant articles; disagreements were resolved by consensus. Given the narrative format of this review, no formal risk-of-bias assessment or meta-analytic synthesis was performed; data were synthesized narratively, with emphasis on mechanistic coherence, clinical relevance, and the consistency of available evidence across preclinical and clinical sources.

Studies were considered eligible for inclusion if they met all of the following criteria:

- **Population/subject:** preclinical models (in vitro receptor studies, rodent and other animal models) or human subjects (healthy volunteers, patients with chronic or acute pain, patients with opioid use disorder, opioid-naïve individuals, pediatric populations, or postmortem cohorts).
- Intervention/exposure: buprenorphine administered by any route (sublingual, buccal, transdermal, intramuscular, intravenous, intranasal, subdermal implant, or extended-release injection), alone or in combination with naloxone or other central nervous system–active substances.
- Outcomes of interest: at least one of the following—receptor binding/affinity at μ-, κ-, δ-, or NOP receptors; G-protein versus β-arrestin signaling bias; respiratory depression and ceiling effect; analgesic efficacy; euphorigenic effects; pharmacokinetic parameters (bioavailability, half-life, metabolism, elimination); drug–drug interactions; precipitated withdrawal; overdose; toxicity; or mortality.
- Study designs: preclinical animal studies, controlled human volunteer pharmacodynamic studies, randomized and non-randomized clinical trials, prospective and retrospective cohort studies, retrospective postmortem and forensic toxicological analyses, poison-center registry studies, case reports, case series, systematic reviews and meta-analyses, and previously published narrative reviews.
- **Language and availability:** publications in English with full text accessible to the authors.
- Time frame: all publications from database inception through March 2026; no lower date limit was applied in order to capture seminal historical work on buprenorphine pharmacology. For most studies, the time frame was the last 5 years.
- Source quality: peer-reviewed journals, together with selected authoritative grey-literature sources (e.g., the WHO World Drug Report, ASAM National Practice Guideline, StatPearls monographs), where they provided clinically relevant epidemiological or guideline information not otherwise available.

Records were excluded if they met any of the following:

- Publications not directly related to buprenorphine pharmacology, toxicology, or clinical use, or in which buprenorphine was mentioned only incidentally.
- Studies focused exclusively on other opioid agonists (e.g., methadone, morphine, fentanyl) without comparative or mechanistic data relevant to buprenorphine.
- Veterinary studies in which findings could not be translated to human pharmacology.
- Articles not available in English or for which the full text could not be retrieved.
- Conference abstracts, posters, editorials, letters to the editor, and commentaries lacking original data or clinically relevant synthesis.
- Duplicate publications and retracted papers.
- Studies considered to be of insufficient methodological quality to support the claims advanced by their authors, as judged by consensus among the reviewers.

A total of 5538 records were identified from PubMed, 20,754 from Embase, and 6901 from Web of Science. After removal of duplicates and initial screening of titles and abstracts, a total of 189 full-text articles were assessed for eligibility. Of these, 111 publications were included in the narrative synthesis, selected based on their relevance to the pharmacology, toxicology, and clinical implications of buprenorphine’s ceiling effect.

## 3. Pharmacokinetics and Pharmacodynamics of Buprenorphine in a Clinical Context

### 3.1. Pharmacology and Medicinal Chemistry

Buprenorphine (C29H41NO4) is a semisynthetic analog of thebaine, a natural alkaloid that can be found naturally in the opium poppy (*Papaver somniferum*). It features a highly complex structure due to its seven stereogenic centers. In terms of synthesis, it follows the classical Bentley approach, which includes several stages of chemical transformation, namely:

1. Diels-Alder reaction between thebaine diene and methyl vinyl ketone.
2. Hydrogenation of the remaining double bond to stabilize the skeleton.
3. Reaction with the Grignard reagent to form a bulky tert-butyl residue.
4. Alkylation reaction, including multiple steps to form a new substituent at the nitrogen atom in the molecule (cyclopropylmethyl).
5. De-methylation thus completes the formation of the desired structure [30,31].

A comprehensive understanding of buprenorphine’s pharmacological properties is essential for recognizing its clinical effects and therapeutic applications. Buprenorphine is an opioid with a distinctive receptor profile. By extension, buprenorphine exhibits unique pharmacological actions. As a partial agonist at the mu-opioid receptor (MOR), it activates the receptor [30]. Still, it produces a submaximal response compared to full agonists, thereby limiting the risk of respiratory depression. At kappa and delta opioid receptors, buprenorphine acts as an antagonist, blocking the effects of endogenous and exogenous agonists [13]. At the same time, certain animal studies have demonstrated inverse agonist activity [31]. This matter remains inconclusive and requires further investigation. Buprenorphine’s activity at the nociceptin/orphanin FQ receptor further enhances its analgesic and antinociceptive properties [13,14,16].

**Figure 1:**
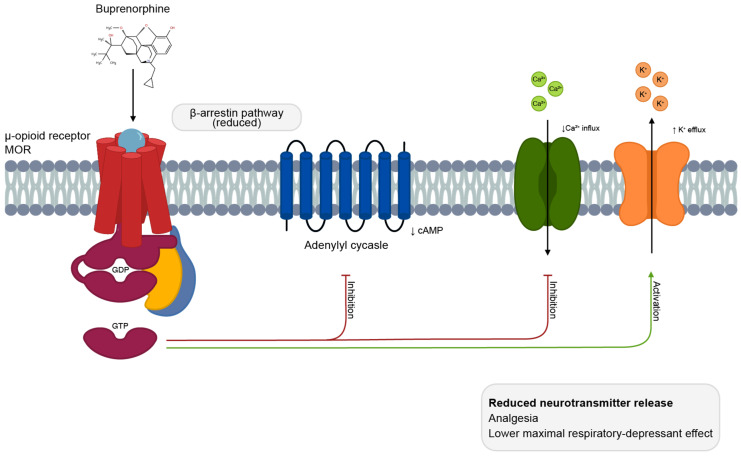
Schematic representation of intracellular signaling mediated by the μ-opioid receptor (MOR). Buprenorphine activates Gi/o signaling, leading to inhibition of adenylyl cyclase, reduced cAMP production, decreased calcium influx, and increased potassium efflux. These effects reduce neurotransmitter release and contribute to analgesia, with a lower maximal respiratory-depressant effect. Figure 1 is an original, author-created schematic illustration prepared specifically for this manuscript. The initial design was created using Procreate (version 5.3.15; Savage Interactive Pty Ltd., Hobart, Australia) and finalized using Adobe Illustrator (version 29.8.7; Adobe Inc., San Jose, CA, USA).

Buprenorphine’s high affinity for opioid receptors, particularly MOR, enables it to displace other opioids and maintain prolonged receptor occupancy [32]. As a biased agonist, it preferentially activates G protein signaling over β-arrestin pathways, a mechanism associated with a reduced incidence of adverse effects such as respiratory depression (Figure 1) [33].

These safety advantages are closely associated with buprenorphine’s unique receptor pharmacology and its ‘ceiling effect’. Buprenorphine is a strong partial agonist of μ-receptors and an antagonist for κ- and δ-receptors. As a consequence of the mild action on the production of euphoria, buprenorphine has a reduced risk of being misused compared to full agonists like morphine or oxycodone. In the therapy of opioid use disorder, it is usually prescribed together with naloxone or serves as an option for methadone, a full agonist of μ-receptor but also an antagonist of NMDA receptors.

Some of the structural analogs discussed in the literature are:

- **Fentanyl analogs:** Highly selective compounds that are 50–100 times stronger in their action compared to that of morphine. This group includes the following: medical drugs (sufentanil, alfentanil, remifentanil); veterinary drug carfentanil; illegal analogs (ocfentanil, furanylfentanil, acrylfentanil) [34,35].
- Benzamide and piperazine derivatives: New synthetic opioids with high toxicity and affinity to μ-receptors, including U-47700 (7.5 times stronger than morphine), AH-7921, and MT-45 [34,35].
- Analogs of plant origin (Kratom): Contains two alkaloids—mitragynine and 7-hydroxymitragynine, which function as partial agonists of the μ-receptors and are considered biased agonists. The main peculiarity of these drugs consists of the absence of β-arrestin recruitment, thus implying low risks of respiratory depression [34,35].
- **Semi-synthetic and non-prescription drugs:**
  - **Desomorphine (“krokodil”): A very dangerous derivative of codeine [34,35].**
  - **Loperamide:** A μ-agonist misused for doses above 200 mg/day to reduce the manifestation of withdrawal symptoms [34,35].

These pharmacological characteristics have significant clinical implications. Buprenorphine’s reduced risk of respiratory depression makes it a safer option for pain management and opioid use disorder in populations at elevated risk for overdose [36,37,38]. In the management of opioid use disorder, buprenorphine is preferred due to its ability to prevent withdrawal symptoms and reduce cravings without inducing euphoria, making it a practical choice in medication-assisted therapy of opioid use disorder [39]. In chronic pain management, its lower risk of causing respiratory depression provides a safety advantage over traditional opioids, particularly for patients with concurrent respiratory conditions [37]. Its strong receptor affinity and slow dissociation decrease the likelihood of relapse in opioid-dependent patients. Furthermore, the limited development of tolerance supports its use in long-term maintenance therapy, underscoring its value in both acute and chronic clinical contexts [38].

### 3.2. Pharmacokinetics

Buprenorphine exhibits poor oral bioavailability, approximately 10–15%, due to extensive first-pass metabolism. Alternative routes of administration that bypass the gastrointestinal tract, such as sublingual, buccal, intranasal, intramuscular, and intravenous, substantially increase bioavailability [40,41,42]. Sublingual administration is preferred in clinical practice for its effective, steady absorption and convenience. Buccal administration is similarly convenient and offers slightly higher bioavailability than sublingual forms. Intravenous administration, which provides complete bioavailability, is generally reserved for acute situations due to its nature [25].

Following absorption, buprenorphine is 96% protein-bound, which limits the concentration of the free drug [13]. Together with its partial agonism and signaling bias, this contributes to a lower side-effect profile compared with full agonists. Partial agonism also mitigates MOR desensitization, reducing the risk of tolerance [12]. The slow dissociation rate from MOR results in sustained receptor occupancy and supports its prolonged duration of action [13]. Buprenorphine displays a relatively long biological half-life and achieves strong receptor occupancy at low plasma concentrations [12,13,14,32].

**Figure 2:**
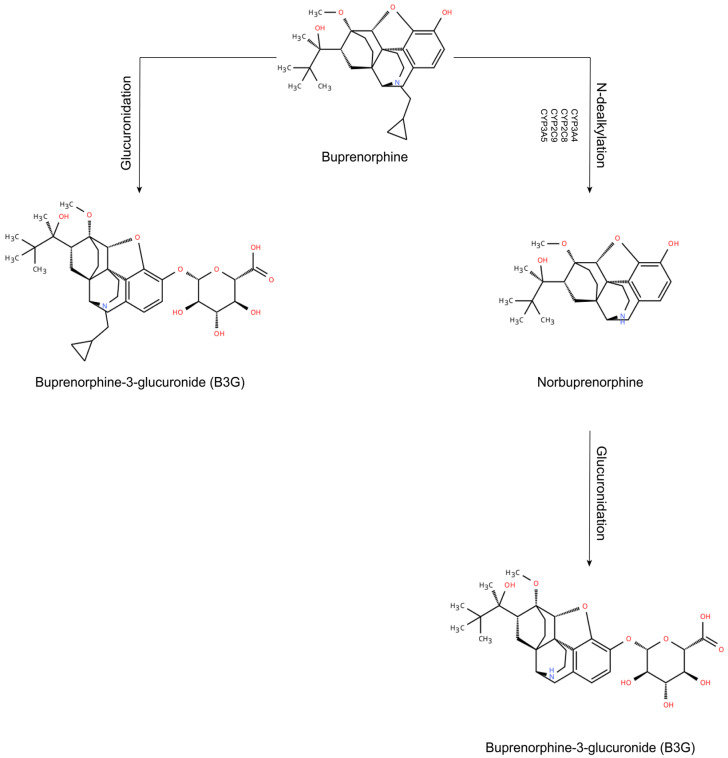
Metabolic pathways of buprenorphine and structures of its primary metabolites. Buprenorphine undergoes glucuronidation to form buprenorphine-3-glucuronide (B3G). Additionally, it is converted to norbuprenorphine by N-dealkylation, mainly via CYP3A4. Norbuprenorphine is further glucuronidated to form norbuprenorphine-3-glucuronide (N3G). Figure 2 is an original, author-created illustration prepared specifically for this manuscript. Chemical structures were generated using MolDraw (web-based molecular structure editor), and the final figure was assembled using Adobe Illustrator (version 29.8.7; Adobe Inc., San Jose, CA, USA).

Buprenorphine undergoes N-dealkylation and glucuronidation, primarily via hepatic cytochrome P450 enzymes, particularly CYP3A4 and, to a lesser extent, CYP2C8, CYP2C9, and CYP3A5 [43]. These metabolic pathways generate three principal metabolites: buprenorphine-3-glucuronide (B3G), N-norbuprenorphine, and norbuprenorphine-3-glucuronide (N3G) (Figure 2).

Buprenorphine-3-glucuronide exhibits minimal pharmacological activity at opioid receptors and contributes little to clinical outcomes. Norbuprenorphine displays higher intrinsic efficacy at MOR than buprenorphine. Full or near-full agonist activity reported in some assays is largely excluded from the central nervous system due to limited blood–brain barrier penetration, resulting in primarily peripheral opioid effects. Norbuprenorphine-3-glucuronide also demonstrates limited central activity; it is highly water-soluble and, therefore, poorly penetrates the blood–brain barrier. Nevertheless, it is considered active [13,44].

Additionally, buprenorphine’s high lipophilicity, low molecular weight, and substantial protein binding (96%) directly influence its potency and pharmacokinetic properties [13]. Those properties cause buprenorphine’s high blood–brain barrier penetration. Understanding these metabolic pathways is crucial for determining dosing strategies, as the limited central activity of norbuprenorphine reduces central opioid effects and influences dose calibration to achieve therapeutic outcomes. This knowledge also supports enhancing safety profiles by minimizing potential side effects and optimizing efficacy, particularly in patients with compromised hepatic function, who may metabolize buprenorphine differently. The main elimination route is through the feces, accounting for over 70%, whereas urination eliminates only up to 30%. Given that CYP3A4 enzymes are the primary pathway for buprenorphine metabolism, it is important to monitor patients taking buprenorphine in combination with inhibitors (ketoconazole) or inducers (rifampicin) [34,35].

In the table below, we compiled all information on the buprenorphine route of administration, dosing, approximate bioavailability, half-life, and clinical applications. Table 1 below compares the listed options by their safety profiles.

## 4. The Ceiling Effect as a Determinant of Safety

The ceiling effect in pharmacology refers to the point on the dose–response curve at which further increases in dose produce no additional therapeutic benefit [49]. In the case of buprenorphine, the ceiling effect, to some extent, is a protective factor against fatal overdoses in monosubstance use. Furthermore, the ceiling effect regarding euphoric properties of buprenorphine does not apply to analgesia [19,25]. Studies involving human volunteers have demonstrated that increasing doses of buprenorphine result in a plateau of adverse effects. At the same time, plasma concentrations continue to rise linearly, indicating a pharmacodynamic ceiling rather than a limitation in absorption [21,27,50]. This partial agonist activity accounts for the ceiling effect observed in respiratory depression and euphoria, as its moderate intrinsic activity triggers limited receptor activation. To clarify, a partial agonist activates receptors, but not to the full extent, leading to a reduced response. At the molecular level, partial agonists like buprenorphine bind to mu-opioid receptors, inducing a degree of receptor activation that is significant but not maximal. Compared with full agonists, which fully activate the receptor and produce a maximal biological response [27].

The ceiling effect on respiratory depression arises from buprenorphine’s pharmacological profile as a partial μ-opioid receptor agonist rather than from saturation of available receptors. Buprenorphine is characterized by very high affinity (it binds strongly to the receptor) but low intrinsic activity at μ-opioid (MOR) receptors. Consequently, even at 100% receptor occupancy, the pharmacological effect it induces reaches a maximum threshold and does not increase further with increasing dose, unlike full agonists [22,51]. Importantly, this ceiling does not extend to analgesia, because sufficient receptor reserve at spinal and supraspinal sites allows submaximal MOR activation to translate into full pain relief [22]. Nevertheless, norbuprenorphine, an active metabolite of buprenorphine, exhibits higher intrinsic efficacy at the MOR than buprenorphine itself and lacks a comparable pharmacological ceiling; this has been proposed as a contributing factor to buprenorphine-related ventilatory failure [44].

Despite the established ceiling effect, there are significant limitations to consider. The ceiling for respiratory depression does not eliminate risk. Patients with compromised respiratory function, opioid-naive patients, or those using other central nervous system depressants concurrently should be under close medical supervision [52,53]. Additionally, individual differences in metabolism, comorbidities, and concomitant medications may influence both efficacy and safety, potentially reducing the protective benefits of the ceiling effect in specific clinical scenarios. Understanding this dosing range enables clinicians to relate study findings to real-world situations, while recognizing that careful patient monitoring remains essential to preserve buprenorphine’s safety profile across various settings.

All clinical indications and dosing are outlined in a table below. Table 2 and Table 3 depict the relationship between the dose and safety among different clinical indications.

Table 2 and Table 3 below demonstrate how the safety of buprenorphine varies by therapeutic indication. It can be observed that, in general, lower doses (<2 mg) are used in the treatment of pain, while much higher doses are used in opioid replacement therapy. Doses that are commonly used in OUD may cause toxicity symptoms in opioid-naive patients.

## 5. Buprenorphine Drug Interactions—Implications in the Context of Polypharmacy and Drug Safety

Polypharmacy often complicates the management of patients receiving buprenorphine because of the risk of significant drug–drug interactions. These interactions can diminish therapeutic efficacy, increase adverse effects, or lead to treatment failure [54,55,56].

### 5.1. Pharmacokinetic Drug Interactions

Notable drug–drug interactions arise, for example, from antiretrovirals that inhibit CYP3A4, potentially elevating or decreasing buprenorphine plasma concentrations [54,55,56]. Regular assessments of liver and kidney function might also be necessary in patients with impaired liver or kidney function, depending on the patient’s overall health status [49].

### 5.2. Pharmacodynamic Drug Interactions

Common interacting substances include benzodiazepines, Z-drugs, and gabapentinoids, which may intensify respiratory depression [54,55,56]. The concurrent use of buprenorphine and benzodiazepines in individuals treated for opioid use disorder is linked to a heightened risk of life-threatening respiratory depression, especially when alcohol is consumed or pre-existing respiratory compromise is present [11,27,51,57,58]. Paradoxically, buprenorphine can also be used to reverse overdoses of other opioids by displacing full mu-receptor agonists, due to buprenorphine’s high receptor affinity [59].

Alcohol and illicit opioids further amplify central nervous system depression [60]. While buprenorphine alone presents a low risk of overdose, combining it with other central nervous system depressants, such as benzodiazepines, Z-drugs, or alcohol, significantly increases the possibility of life-threatening respiratory depression by surpassing the ceiling effect that limits typically adverse respiratory outcomes. These intricate pharmacodynamic interactions require vigilant monitoring and frequent adjustment of treatment regimens to achieve safer, more effective outcomes [24,61].

In a broader clinical context, there were several clinical cases where concurrent use of serotonergic antidepressants and buprenorphine caused serotonin syndrome [62,63]. A proposed mechanism involves modulation of monoaminergic transmission via KOR antagonism, although direct evidence linking buprenorphine to serotonin syndrome remains limited and largely confined to case reports involving polypharmacy [64]. The second reason is that in the aforementioned studies, the cases described were unusual because they involved the use of two or more serotonergic medications and/or central nervous system (CNS) depressants. The exact mechanism in humans remains unknown, and further research is needed.

### 5.3. Interactions with Prescription Drugs

The CDC advises using the lowest effective dose for the shortest effective duration when combining opioids with other CNS depressants. When co-prescribing cannot be avoided, close monitoring of the patient’s respiratory function is crucial [46].

### 5.4. Interactions with Other Unrelated Drugs

The use of illicit substances introduces additional complexity because of their unpredictable pharmacological profiles and potential interactions with prescribed medications. Those substances increase the risk of overdose or withdrawal when stopped abruptly [24].

### 5.5. Clinical Recommendations, Monitoring, and Safety Management

To manage these risks, initial steps should include considering dose reduction and enhanced monitoring. This approach helps specialists quickly translate identified risks into practical strategies [65]. When illicit substance use is suspected or confirmed, clinicians should consider reducing buprenorphine doses to mitigate the risk of respiratory depression [56].

Monitoring should include specific parameters such as respiratory rate, mental status, and signs of withdrawal when buprenorphine is used for substance use disorder. Validated assessment tools, such as the Clinical Opiate Withdrawal Scale (COWS) and the brief mental status evaluation, can be used to monitor physical and mental conditions precisely [66,67]. COWS helps in determining the severity of opioid withdrawal symptoms, guiding appropriate dosing of buprenorphine [67]. The Brief Mental Status evaluation provides insights into a patient’s cognitive functioning and potential mental health issues that require attention.

It was determined that buprenorphine alone is associated with a very low risk of overdose [68]. For clinical practice, it is recommended that healthcare providers emphasize the importance of avoiding concurrent use of buprenorphine with other CNS depressants, unless necessary. Monitoring steps should include regular checks of respiratory rate, pulse oximetry to measure oxygen saturation, and observing for signs of respiratory distress such as shortness of breath or cyanosis. To manage these risks, doctors must understand and implement effective dose adjustment strategies. Healthcare professionals should also conduct thorough assessments and implement ongoing monitoring to prevent adverse effects according to the 2020 ASAM National Practice Guideline for the Treatment of Opioid Use Disorder [69].

It is also important to educate patients on recognizing signs of overdose, such as difficulty breathing or unresponsiveness, and to ensure they understand the importance of seeking emergency help promptly. Regular follow-ups can mitigate risks.

These challenges underscore the importance of thorough medication review and interdisciplinary collaboration. Comprehensive clinical strategies are required to optimize patient safety and therapeutic outcomes in buprenorphine therapy. Effective management relies on an integrated approach that anticipates and addresses these complexities, thereby ensuring both efficacy and safety in clinical practice.

## 6. Can Buprenorphine Be Overdosed—Case Study Analysis

### 6.1. Buprenorphine Overdose Symptoms

Clinical and preclinical studies demonstrate that buprenorphine possesses a superior safety profile relative to other opioids, primarily due to its partial agonist properties and the ceiling effect on respiratory depression. However, despite a lower overall risk of fatal overdose compared to full opioid agonists such as morphine or methadone, buprenorphine still presents significant risks. Fatal overdose may occur under specific circumstances, particularly among vulnerable populations, including pediatric patients, opioid-naive individuals, and those concurrently using other central nervous system depressants, each with distinct risk factors.

Buprenorphine overdose significantly impacts the central nervous, respiratory, and cardiovascular systems. Although the symptoms of buprenorphine toxicity are similar to those observed in overdoses of other opioids, the severity is generally reduced due to its ceiling effect [70,71]. Common manifestations include central nervous system depression, sedation, dizziness, confusion, bradypnea, apnea, bradycardia, hypotension, nausea, vomiting, and miosis [17].

### 6.2. Buprenorphine Overdose and Toxicity in Animal Models

The LD50 of buprenorphine has not been determined in human studies, limiting the strength and direct applicability of the available evidence. In animal studies, the LD50 for intravenous buprenorphine was determined to be 24 mg/kg in mice and 31 mg/kg in rats [72]. In mice, the LD50 of oral buprenorphine is 260 mg/kg [72]. However, these data are drawn from older sources and animal research, which have inherent limitations due to differences in species-specific metabolism and physiology. Animal models directly validate the human pharmacodynamic ceiling effect, with escalating buprenorphine doses plateauing in respiratory effects [22,73]. Cases of animal studies determined that norbuprenorphine, a metabolite of buprenorphine, is a powerful driver of respiratory depression, due to the lack of its ceiling effect [74]. The average LD50 of norbuprenorphine is 10 mg/kg, which is much lower than the LD50 of buprenorphine [75]. The apparent cause of death was deep coma and apnea [75].

Furthermore, animal studies demonstrated that the ceiling effect is effectively abolished by co-administration of alcohol and benzodiazepines, through synergistic pharmacokinetic depression of ventilation [76,77,78].

Animal studies provide valuable insight into the mechanistic actions of buprenorphine, yet no single animal study captures the mechanism of human buprenorphine overdose. Interspecies differences in metabolism and receptor pharmacology. Animal LD50 values cannot be used to precisely estimate the lethal dose in humans.

### 6.3. Buprenorphine Overdose and Poisoning in the Pediatric Population

The pediatric population is particularly vulnerable to buprenorphine toxicity, necessitating focused consideration of the unique risks and factors contributing to adverse outcomes in children and adolescents. These vulnerabilities are influenced by both social and biomedical factors, including differences in drug metabolism due to physiological characteristics, presumed opioid naivety, and the high prevalence of accidental exposure. The most affected subgroups are children under seven years of age, typically due to unintentional ingestion, and adolescents, often in the context of substance use disorder or suicide attempts [79,80]. In children under seven, doses below 2 mg pose a moderate risk and may result in severe symptoms such as lethargy and miosis, though the probability of severe outcomes remains low. Doses between 2 mg and 4 mg present a moderate risk of serious harm, with potential for severe central nervous system depression. Doses exceeding 4 mg are considered potentially fatal, with universal toxicity and a high risk of respiratory arrest [81]. The ToxIC NOSE (Novel Opioid and Stimulant Exposure) initiative classifies buprenorphine as a “one pill can kill” substance [82,83]. For example, a case of accidental exposure involved a child using a buprenorphine transdermal patch during sibling play, resulting in severe toxicity due to the patch’s harmless appearance [84]. To mitigate these risks, preventive strategies are essential, including secure medication storage and caregiver education regarding the dangers of buprenorphine exposure. Keeping medications out of children’s reach and equipping caregivers to recognize early signs of toxicity can substantially reduce accidental exposures and improve emergency response.

### 6.4. Buprenorphine Poisoning in Opioid-Naïve Population

In opioid-naive individuals, fatal buprenorphine overdose without polysubstance use is extremely rare. However, severe respiratory depression and toxicity remain possible. In this group, buprenorphine toxicity is most often iatrogenic, typically resulting from rapid initiation or administration of long-acting formulations [85]. A review of postmortem toxicological studies found that, over a seven-year period, only 4 of 55 buprenorphine-related deaths did not involve other substances. In these cases, the cause of death was suspected to be unrelated to buprenorphine use [24].

### 6.5. Buprenorphine-Related Deaths in Cases of Polysubstance Use

The majority of buprenorphine-related deaths are associated with polysubstance use [86,87]. Retrospective postmortem toxicological analyses consistently indicate that buprenorphine toxicity is rarely the sole cause of death; most cases involve additional substances such as other opioids, benzodiazepines, gabapentinoids, psychostimulants, cannabinoids, or alcohol [24,26,88]. Certain combinations, particularly with benzodiazepines or alcohol, are especially hazardous due to their synergistic depressant effects on the central nervous system, which markedly increase the risk of respiratory depression and fatality. Recognizing these patterns is essential for risk assessment and for developing preventive strategies targeting health outcomes related to buprenorphine and polysubstance use. Research into fatal cases involving buprenorphine and polysubstance use is limited by methodological challenges; ethical constraints necessitate reliance on postmortem toxicological analyses and animal studies. The generalizability of animal research is limited by interspecies differences in physiology and metabolism.

In contrast, postmortem analyses can detect metabolites but cannot determine the precise ingested dose or account for individual metabolic variability. Despite these limitations, such research provides valuable insights into the mechanisms underlying buprenorphine-related deaths. For example, an animal study demonstrated that the combination of buprenorphine and diazepam induced earlier onset of sedation and respiratory depression than either drug alone [76]. This effect may be explained by a benzodiazepine-mediated increase in brain buprenorphine concentrations (pharmacokinetic hypothesis) and their combined impact on respiratory function [76,77]. Another animal study had demonstrated the synergistic effect of buprenorphine and ethanol on respiration [78]. The relationship between buprenorphine dose and lethal doses of other substances appears to be correlational, as animal studies offer incomplete evidence, and in postmortem studies, buprenorphine or norbuprenorphine levels are often within therapeutic or slightly supratherapeutic ranges alongside potentially fatal doses of other drugs. The synergistic effect of buprenorphine and other CNS depressants is well demonstrated in animal studies. In conclusion, while polysubstance use is the predominant factor in buprenorphine-related fatalities, current research is limited by methodological constraints, highlighting the need for further rigorous studies to clarify the complex interactions and risk factors involved.

## 7. Buprenorphine in Opioid Overdose Management

Buprenorphine possesses a unique receptor profile and an established ceiling effect, characteristics that make it potentially valuable in treating overdoses involving other opioids. Although naloxone remains the gold standard for reversing acute opioid overdose, buprenorphine may be beneficial in select cases [23]. Following any suspected buprenorphine exposure, patients should be closely monitored for these symptoms. Initial management steps involve ensuring airway support and administering naloxone as a reversal agent [66]. In severe cases, caution should be taken due to potential incomplete reversal of buprenorphine-related respiratory depression [89]. This limitation is due to buprenorphine’s high affinity for opioid receptors, which may prevent naloxone, even at high doses, from entirely displacing it. As a result, clinical management should include higher doses of naloxone, extended monitoring, and supportive care [89]. To effectively initiate buprenorphine post-overdose, it is crucial to consider both the timing and the patient’s clinical status. Initiation is typically recommended when a patient experiences mild to moderate withdrawal symptoms, indicated by a Clinical Opiate Withdrawal Scale (COWS) score of 12 or higher [67]. This protocol helps minimize the risk of precipitated withdrawal and ensures optimal patient selection. As a maintenance therapy for opioid use disorder, buprenorphine has been associated with lower mortality rates compared to methadone [90]. Its high receptor affinity and ceiling effect on respiratory depression further support its utility in both the prevention and treatment of opioid overdose [51,91]. Initiating buprenorphine in emergency settings can bridge the gap between acute overdose management and opioid replacement therapy, thereby reducing treatment discontinuity and improving patient adherence [92,93,94]. As a result, overall mortality is significantly reduced.

Although buprenorphine is a valuable intervention in opioid overdose management, several important limitations remain. One significant limitation is its reduced efficacy in cases of polysubstance use. When other central nervous system depressants, such as benzodiazepines, are involved, buprenorphine does not adequately mitigate the risk of severe respiratory depression due to pharmacodynamic interactions [77]. Additionally, the presence of high-potency synthetic opioids may diminish buprenorphine’s protective effects, necessitating higher doses to prevent breakthrough cravings [95,96,97]. Therefore, while buprenorphine is an essential tool in opioid overdose treatment, it is important to recognize these limitations and tailor therapeutic strategies to individual patient contexts to optimize outcomes.

## 8. Discussion

Buprenorphine, due to its pharmacodynamic profile, occupies a distinct position in the opioid pharmacopeia. As a semisynthetic derivative of thebaine and a partial μ-opioid receptor agonist, it offers a more favorable respiratory safety profile than traditional full μ-opioid receptor agonists.

Compared with methadone, heroin, morphine, and fentanyl, buprenorphine has a lower maximal respiratory depressant effect, resulting in a wider margin before apnea and death occur [13,51]. Despite its toxicological importance, buprenorphine should not be framed as entirely risk-free. Toxicity can still lead to significant clinical complications even without a fatal outcome, with manifestations ranging from milder symptoms like dizziness and vomiting to severe respiratory compromise, including apnea, bradycardia, and reduced consciousness [98]. Buprenorphine is therefore considered safer in overdose than full opioid agonists concerning respiratory depression, but this protection remains conditional and incomplete [99].

The lower risk of fatal overdose is primarily explained by its partial μ-opioid receptor agonism. With increasing doses, respiratory depression reaches a plateau rather than progressing further, consistent with the ceiling effect observed in human pharmacodynamic studies [51]. Due to its high affinity, buprenorphine effectively occupies receptors without initiating the progressive respiratory depression seen with full μ-opioid receptor agonists [13]. Furthermore, evidence indicates that plasma concentrations can increase independently of the respiratory plateau, suggesting a pharmacodynamic basis for the ceiling effect [13].

Despite these pharmacodynamic advantages, death in buprenorphine overdose, as in other opioids, is still most related to ventilatory failure. The ceiling effect reduces the possibility of respiratory collapse under certain conditions, but it does not eliminate the risk of severe or fatal respiratory toxicity [25,100]. Norbuprenorphine, lacking the same protective characteristics, may contribute to ventilatory failure related to buprenorphine toxicity, although its independent clinical role remains uncertain [44].

The clinical significance of buprenorphine toxicity differs considerably across patient groups. In children, even small absolute exposures may cause clinically significant toxicity in response to low body weight, opioid naivety, and frequent accidental ingestion. Hayes et al. reported toxicity in 54 of 86 pediatric exposures, risk increased at ingestions of 2 mg or more, and toxicity was reported in all children ingesting more than 4 mg [81]. In adult patients, the main risk concerns opioid-naive individuals and those with pre-existing respiratory compromise. Available data also suggest that fatal buprenorphine toxicity in adults rarely reflects isolated exposure. In del Pozo et al., only 4 of 55 buprenorphine-involved deaths did not include other substances, and even in those cases, buprenorphine was not clearly established as the direct cause of death [24]. Wightman et al. identified buprenorphine and norbuprenorphine in 29 of 534 opioid-involved deaths, while only one case listed buprenorphine as the sole implicated drug [92]. These findings suggest that the greatest adult risk is concentrated in patients with low opioid tolerance, impaired respiratory reserve, or medically inappropriate exposure, and most importantly, polysubstance use [101].

Even with these minimal overdose-related risks, contemporary literature indicates that buprenorphine remains one of the most valuable treatments for opioid use disorder. Its high receptor affinity, long-lasting occupancy, and partial agonist profile provide a unique balance between suppressing cravings and preventing fatal overdose [13,51]. This pharmacological profile supports buprenorphine’s central role in maintenance treatment and also explains its value in overdose-related care, particularly as a means of linking acute overdose management with longer-term OUD treatment [48,102]. Nevertheless, widespread fentanyl exposure has made buprenorphine initiation more clinically composed. The same high-affinity receptor pharmacology that supports maintenance treatment and overdose prevention may also precipitate withdrawal when fentanyl or other high-potency full agonists remain present at the receptor [25]. Jones et al. reported withdrawal in 31% of low-dose initiations among outpatients using fentanyl daily, although most cases were mild, and precipitated withdrawal was uncommon without protocol deviation [100]. In addition, Thakrar et al. found precipitated withdrawal in 11.5% overall and in 16.3% of patients with confirmed fentanyl exposure in hospital settings [103]. Buprenorphine therefore retains clear clinical value in OUD treatment and overdose-related care, but initiation after recent fentanyl exposure requires more cautious and individualized induction strategies [95].

Polysubstance use is a major cause of buprenorphine toxicity. The use of buprenorphine with other central nervous system depressants, most commonly including benzodiazepines and alcohol, dramatically increases the chances of an unfavorable outcome. Observational data provide that buprenorphine treatment days were associated with lower odds of drug-related poisoning, despite benzodiazepine or Z-drug treatment increasing poisoning risk, particularly at higher doses [104]. Post-mortem studies reported that the respiratory ceiling associated with buprenorphine does not reliably protect ventilation once additional sedatives further impair ventilatory drive, arousal, and airway protection. In Del Pozo et al., 92.7% of buprenorphine-involved deaths included other potent substances [24]. Wightman et al. found that buprenorphine was rarely the sole implicated drug in opioid-involved fatalities, whereas Mariottini et al. reported that additional depressants were frequently present in buprenorphine-related deaths [26,94,99]. A possible pharmacodynamic explanation for this pattern was proposed by Vodovar et al., who showed that co-exposure to diazepam and buprenorphine accelerated sedation and respiratory depression in animals [76].

However, several important uncertainties remain. Postmortem studies cannot reliably reconstruct the exact dose, timing of ingestion, sequence of exposure, or the individual contribution of each drug to respiratory failure in mixed-drug deaths. Interpretation is further limited by postmortem redistribution and interindividual variability in metabolism. Animal studies provide useful mechanistic insights, especially regarding drug interactions and maximal doses. Still, they do not reproduce human tolerance, comorbidities, or the broader clinical complexity of overdose due to differences in metabolism between species. The maximum tolerated dose also remains unsettled, especially in opioid-tolerant adults. Controlled human toxicity studies are not possible due to ethical reasons. Fatal cases rarely involve buprenorphine alone. The reason behind this phenomenon might stem from social causes associated with opioid use disorder, as buprenorphine is most commonly used in opioid substitution treatment. Mechanistic understanding is similarly incomplete for some interactions, including the extent to which benzodiazepines or alcohol, due to a synergistic effect, merely add respiratory burden or functionally overcome buprenorphine’s protective ceiling [25].

**Figure 3:**
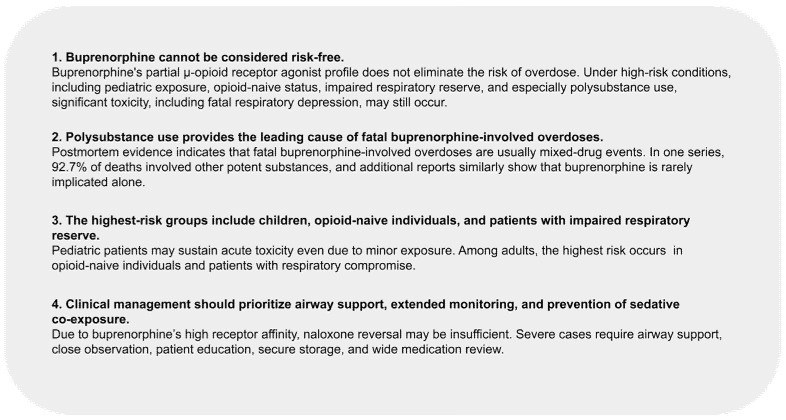
Key takeaways from this manuscript.

Overall, buprenorphine is safer than full opioid agonists in terms of respiratory depression, but it is not risk-free. Its partial agonism and ceiling effect confer a real toxicological advantage over full opioid agonists. Yet, that advantage is reduced in vulnerable patients and may be substantially attenuated by sedative co-exposure. Special attention should be paid to children, opioid-naive individuals, patients with pre-existing respiratory compromise, and those using benzodiazepines, alcohol, or other CNS depressants. In clinical practice, acute assessment should remain focused on ventilation and level of consciousness, especially because naloxone reversal may be incomplete and prolonged monitoring may be required in severe cases [82]. The most important next steps for research are to distinguish isolated buprenorphine toxicity from mixed-drug fatalities more precisely, to clarify the mechanisms of interaction with sedative co-exposure, and to define safer induction strategies in fentanyl-exposed populations. These issues will determine how far buprenorphine’s respiratory safety advantage extends under current real-world conditions (Figure 3).

## Figures and Tables

**Table 1:** The relationship between dosing, route of administration, and safety.

Context	Route of Administration	Dosage Range	Bioavailability (Approx.)	Half-Life (t1/2)	Safety & Clinical Characterization	References
Therapeutic	Transdermal	5–80 µg/h	~15%	24–48 h (after patch removal)	Highest safety margin. Constant plasma levels; avoids “peaks/valleys.” Ideally suited for the elderly.	[42]
Therapeutic	Sublingual	2–32 mg	30–51%	24–69 h	Partial agonist ceiling. Prevents lethal respiratory depression in isolation. Risk: precipitated withdrawal.	[42]
Therapeutic	Buccal film	75–900 mcg	46–65%	24–48 h	Enhanced mucosal delivery. Offers superior bioavailability and consistency compared with sublingual. Risk of localized tissue irritation.	[40,45]
Therapeutic	Subdermal Implant	~80 mg/insert	High (Steady state)	4–6 months	Long-term stability. Constant low-level release; surgical risks (migration/infection) require oversight.	[45]
Therapeutic	Intramuscular	0.3–0.6 mg	90–100%	2–5 h	Predictable analgesia. Rapid onset for acute pain without the complexity of IV access.	[45]
Therapeutic	Intravenous	0.3–0.6 mg	100%	2–3 h	Immediate potency. Requires continuous hemodynamic monitoring due to rapid-onset sedation.	[46]
Supratherapeutic	Acute Over-ingestion	>32 mg (SL)	~30%	Extended	Plateau effect. Receptors saturate at ~32 mg. Respiratory suppression typically plateaus.	[47,48]
Supratherapeutic	Chronic High-Dose	>32 mg	Variable	Accumulative	Saturability. Marginal clinical gain beyond 32 mg; increased risk of hepatic enzyme elevation.	[47]
Illicit	Intranasal (Crushed)	Variable	40–50% *	~24–42 h	Erratic absorption. Bypasses intended release; excipients cause mucosal and pulmonary damage.	[41]
Illicit	I.V. Misuse	Variable	100%	2–3 h	Acute risk transition. Injection of film/tablet triggers precipitated withdrawal if naloxone is present.	[47]
Illicit	Polydrug Use	N/A	N/A	Variable	Lethal Synergism. The ceiling effect is abolished when combined with benzodiazepines or alcohol.	[47]

Note: SL = sublingual; IV = intravenous; N/A = not applicable; μg/h = micrograms per hour; mcg = micrograms; mg = milligrams. * bioavailbility cannot be precisely determined due to lack of precise pharmacological data.

**Table 2:** Buprenorphine dose, receptor occupancy, and risk of respiratory depression (sublingual equivalent doses). Receptor occupancy values and safety characterizations derived from Grande et al. [43], Miller et al. [53], and Nielsen et al. [20].

Dose (Sublingual Equivalent)	Receptor Occupancy (μOR)	Respiratory Depression Risk	Clinical Safety Notes	Refs.
Micro-dose (≤1 mg)	≤50%	Minimal	High safety margin. Minimal sedation or respiratory risk across all populations.	[44,51]
Low dose (2–4 mg)	~40–60%	Moderate (opioid-naïve)	Danger: toxicity risk for non-tolerant adults. Ceiling effect not yet established at this range.	[52]
Moderate dose (8–16 mg)	~70–85%	Plateau reached (ceiling effect)	Safety ceiling: respiratory depression plateaus at this dose range. Standard FDA target for OUD (16 mg).	[43,53]
High dose (24 mg)	~90%	Plateau maintained	Ceiling effect preserved. Associated with 20% better health outcomes compared to 16 mg.	[43,53]
High therapeutic dose (32 mg)	~95–98%	Plateau; lethal risk in opioid-naïve	Respiratory side effects plateau. 50% better OUD retention vs. 16 mg in 2024 studies. Potentially lethal in non-tolerant individuals.	[43,53]
Supratherapeutic (>40 mg)	~100%	Variable; toxicity possible	Doses up to 44 mg/70 kg demonstrated as safe in controlled studies. Risk increases with polysubstance use.	[20,43]

Note: μOR = μ-opioid receptor; OUD = opioid use disorder; FDA = Food and Drug Administration; mg = milligrams.

**Table 3:** Buprenorphine dosing by clinical indication (sublingual equivalent doses). Dosing recommendations are derived from Grande et al. [43], Miller et al. [53], Adams et al. [51,52], and Zimmerman et al. [44].

Dose (Sublingual Equivalent)	Chronic Pain (Opioid-Naïve)	Acute Pain (ER/Post-Op)	Opioid Use Disorder (OUD)	Refs.
Micro-dose (≤1 mg)	Standard: effective at 150–900 mcg (buccal/transdermal formulations)	Standard: 0.3 mg IV/IM every 6 h	Micro-induction: the “Bernese Method”; low-dose initiation without prior withdrawal	[44,51]
Low dose (2–4 mg)	Supratherapeutic: high risk of toxicity in opioid-naïve patients	Standard: high standard acute dose	Subtherapeutic: insufficient to block cravings or prevent withdrawal	[52]
Moderate dose (8–16 mg)	—	—	Standard target: current FDA target dose (16 mg); effective for craving suppression	[43,53]
High dose (24 mg)	—	—	Above-standard: used for prevention of fentanyl/heroin cravings; 20% better health outcomes vs. 16 mg	[43,53]
High therapeutic dose (32 mg)	Contraindicated: potentially lethal in non-tolerant individuals	—	High-dose induction: used to stabilize high-potency fentanyl users; 50% better retention vs. 16 mg	[43,53]
Supratherapeutic (>40 mg)	—	—	ER/refractory OUD: may cause toxicity; doses up to 44 mg/70 kg shown safe in controlled settings	[20,43]

Note: “—” indicates the dose is not applicable or not recommended for that indication. Micro-dose chronic pain dosing typically uses transdermal (5–80 µg/h) or buccal formulations (75–900 µg) rather than sublingual. OUD = opioid use disorder; ER = emergency department.

## Data Availability

No new data were created or analyzed in this study.
